# A Bidirectional Analysis of Inflammatory Bowel Disease and Gout: Epidemiologic Evidence of a Stronger Association in Crohn’s Disease from a Nationwide Cohort Study in South Korea

**DOI:** 10.3390/biomedicines14030613

**Published:** 2026-03-09

**Authors:** Dae Myoung Yoo, Hyo-Jeong Lee, Ho Suk Kang, Hyo Geun Choi, Kyeong Min Han, Joo Hee Kim, Woo Jin Bang, Mi Jung Kwon

**Affiliations:** 1 Hearing, Balance and Integrated Neuroscience Laboratory, Hallym University College of Medicine, Anyang 14068, Republic of Korea; ydm1285@naver.com (D.M.Y.); or km_han@hallym.ac.kr (K.M.H.); 2 Hallym Data Science Laboratory, Hallym University College of Medicine, Anyang 14068, Republic of Korea; 3 Department of Otorhinolaryngology-Head and Neck Surgery, Hallym University Sacred Heart Hospital, Hallym University College of Medicine, Anyang 14068, Republic of Korea; 4 Division of Gastroenterology, Department of Internal Medicine, Hallym University Sacred Heart Hospital, Hallym University College of Medicine, Anyang 14068, Republic of Korea; hskang76@hallym.or.kr; 5 Suseo Seoul E.N.T. Clinic, 10 Bamgogae-ro 1-gil, Gangnam-gu, Seoul 06349, Republic of Korea; mdanalytics@naver.com; 6 Division of Pulmonary, Allergy and Critical Care Medicine, Department of Medicine, Hallym University Sacred Heart Hospital, Hallym University College of Medicine, Anyang 14068, Republic of Korea; luxjhee@hallym.or.kr; 7 Department of Urology, Hallym University Sacred Heart Hospital, Hallym University College of Medicine, Anyang 14068, Republic of Korea; 8 Department of Pathology, Hallym University Sacred Heart Hospital, Hallym University College of Medicine, Anyang 14068, Republic of Korea

**Keywords:** inflammatory bowel disease, Crohn’s disease, ulcerative colitis, gout, bidirectional study, nationwide cohort

## Abstract

**Background:** Inflammatory bowel disease (IBD) and gout are both chronic inflammatory conditions. Emerging evidence suggests a potential link between IBD and gout through shared inflammatory and metabolic pathways; however, epidemiological findings remain limited and inconsistent, and the temporal direction of this association has not been clearly established. **Methods:** Through the use of the South Korean National Health Insurance database, we conducted a nationwide bidirectional matched cohort study. In Study I, 10,793 patients with IBD were matched 1:4 to controls without IBD. In Study II, 25,624 patients with gout were matched 1:4 to nongout controls. Exact matching was performed on age, sex, income level, and region of residence, followed by propensity score overlap weighting. Overlap-weighted Cox proportional hazards models were employed to measure adjusted hazard ratios (aHRs) for incident gout (Study I) and incident IBD (Study II). **Results:** In Study I, patients with IBD experienced a greater occurrence of gout than matched controls (28.40 vs. 24.20 per 10,000 person-years). IBD was linked to a higher likelihood of gout (aHR, 1.16; 95% CI, 1.04–1.29). When stratified by subtype, Crohn’s disease (CD) revealed a significant relationship with gout (aHR, 1.25; 95% CI, 1.05–1.48), whereas ulcerative colitis (UC) did not. Notably, the association between IBD (including CD and UC) and gout was consistently significant among participants younger than 45 years. In Study II, gout was not related to an elevated likelihood of IBD (aHR, 1.04; 95% CI, 0.91–1.21) or its subtypes. **Conclusions:** IBD, particularly CD, was linked to a greater likelihood of gout, especially among younger adults, whereas gout was not associated with subsequent IBD, suggesting an asymmetric association between IBD and gout.

## 1. Introduction

Inflammatory bowel disease (IBD), which includes Crohn’s disease (CD) and ulcerative colitis (UC), is a chronic immune-mediated disorder of the gastrointestinal tract and is frequently accompanied by extraintestinal complications and reduced quality of life [1,2]. UC is typically characterized by continuous mucosal inflammation extending proximally from the rectum, whereas CD presents with transmural, discontinuous inflammation that may affect any segment of the gastrointestinal tract [3]. Both conditions follow a relapsing–remitting course and commonly manifest as abdominal pain, diarrhea, and hematochezia [1,4]. Extraintestinal manifestations occur in up to half of patients with IBD [2], and arthritis is among the most common and clinically significant of these complications [5,6].

Although IBD was historically considered a disease largely confined to Western countries [1], its incidence has increased rapidly across the Asia-Pacific region, including South Korea [4]. This rise has been attributed, at least in part, to rapid urbanization, industrialization, and shifts toward Western dietary patterns [7]. At the same time, the age distribution of IBD is changing. While disease onset most often occurs in young adulthood, the number of older patients with IBD continues to grow, and it has been estimated that nearly one-third of individuals with IBD will be older than 60 years within the next decade [8]. In Korea, IBD first emerged in the 1980s, followed by a period of accelerated growth, and has now entered a phase of sustained and compounding prevalence [9]. Long-term data from Seoul demonstrated marked increases in the incidence of both CD (from 0.06 to 2.44 per 100,000) and UC (from 0.29 to 5.82 per 100,000) over three decades, reaching levels that exceed those reported in several neighboring Asian countries (Japan, Hong Kong, or India) [4,7].

Gout, the most common form of inflammatory arthritis, develops in the setting of persistent hyperuricemia and the deposition of monosodium urate crystals in joints [10]. However, its pathogenesis is not solely a matter of urate accumulation. Crystal-induced activation of the NLRP3 inflammasome promotes interleukin (IL)-1β production and triggers recurrent inflammatory flares with systemic involvement [11]. Gout is frequently accompanied by comorbid conditions such as hypertension, chronic kidney disease, and metabolic syndrome [12,13]. In Korea, the prevalence of gout has risen sharply—from 0.39% in 2002 to 2.01% in 2015—representing a 5.15-fold increase and an average annual increase of 10.8% in gout-related healthcare costs [14]. This represents a greater increase than that observed in other countries; the prevalence of gout increased 1.64-fold in the United Kingdom between 1997 and 2012 [15], 1.4-fold in the United States between 1988–1994 and 2007–2008 [16], and 1.12-fold in Taiwan between 2005 and 2010 [17,18].

Accumulating evidence suggests a potential biological overlap between IBD and gout. Both conditions involve dysregulated inflammatory pathways in which cytokines such as IL-1β and tumor necrosis factor (TNF)-α play central roles [11,19]. In IBD, macrophages and dendritic cells in the intestinal mucosa respond to Toll-like receptor (TLR) signaling and produce high levels of IL-1β and TNF-α [19], whereas in gout, monosodium urate crystals activate the NOD-like receptor protein 3 (NLRP3) inflammasome, inducing IL-1β production and contributing to chronic inflammation together with TNF-α [11,19]. These shared immune pathways provide a plausible mechanistic link between the two diseases [11,19]. Microbiome-focused studies further support a potential link: Chiaro et al. demonstrated that specific commensal organisms, including *Saccharomyces cerevisiae*—which participates in purine metabolism—were enriched in IBD patients, potentially contributing to elevated uric acid levels [20]. Further animal studies by Lv et al. and Xu et al. indicated that intestinal barrier disruption and increased abundance of inflammatory microbiota upregulated TLR2/4/5 signaling, enhanced IL-1β and TNF-α production, and increased systemic inflammation in hyperuricemic mice [21,22], indicating a potential rise in uric acid levels in IBD patients [23]. Together, these findings raise the possibility that chronic intestinal inflammation, immune response, and gut dysbiosis may influence systemic uric acid metabolism and, in turn, gout risk.

Epidemiologic findings, however, have been inconsistent. A study from the United States reported a higher prevalence of gout among patients with IBD (4.3% in UC and 5.6% in CD) compared with that in the general population (3.5%) [24]. This study demonstrated a meaningful elevation in the risk of gout in patients with IBD, reporting that CD was related to incident gout (OR 1.68, 95% CI: 1.60–1.75) and that UC was related to gout (odds ratio (OR) 1.38, 95% confidence interval (CI): 1.31–1.44) [24]. However, that investigation primarily evaluated the prevalence of gout among patients with IBD using a multicenter electronic health record database and a cross-sectional analytical framework, without assessing temporal directionality or the reverse association between gout and subsequent IBD [24]. A retrospective case–control study in China reported that individuals in the highest quartile of serum uric acid levels had a significantly increased risk of UC (OR = 1.62, 95% CI: 1.24–2.55) [25]; however, this study included only the UC group, and further validation for CD is necessary. In contrast, a population-based cross-sectional study from Switzerland revealed no significant difference in gout incidence between IBD patients and matched controls (OR 1.05, 95% CI: 0.82–1.35) [26]. These inconsistent findings suggest that the IBD–gout association may vary by population or study design. However, most prior studies have focused exclusively on the risk of gout in patients with IBD, whereas evidence addressing the reverse association—specifically whether gout predisposes individuals to developing IBD subtypes—is scarce. Given that the prevalence of both diseases has risen substantially worldwide [1,13], clarifying their potential bidirectional association has important implications for clinical practice and public health [26].

Thus, we hypothesized that IBD and gout may have a reciprocal association. To examine this assumption, two complementary population-based longitudinal studies were planned in a nationwide cohort. Study I evaluated the likelihood of incident gout in patients with IBD compared with matched controls, whereas Study II assessed whether patients with gout were at increased likelihood of subsequent IBD development.

## 2. Materials and Methods

### 2.1. Study Design and Data Source

This retrospective cohort study was conducted using data from the Korean National Health Insurance Service–National Sample Cohort (KNHIS-NSC) [27]. The database includes 1,137,861 individuals and 219,673,817 insurance claims recorded from January 2002 through December 2019. The KNHIS-NSC was established through systematic sampling to generate a nationally representative cohort of 1,025,340 individuals, corresponding to approximately 2.2% of the South Korean population in 2002. Participants were longitudinally followed for up to 17 years, through the end of 2019. Further details regarding the representativeness and structure of the cohort have been described in previous studies [28,29]. This study was approved by the Institutional Review Board of Hallym University (IRB No. 2022-10-008; approved 25 October 2022).

### 2.2. Diagnostic Criteria

IBD was identified by the presence of CD (ICD-10: K50) or UC (ICD-10: K51). Individuals with at least one of these diagnoses were considered to have IBD. Gout was ascertained based on at least two outpatient or inpatient claims carrying a diagnosis of gout (ICD-10: M10). These case definitions were adapted from previously published studies [30,31].

### 2.3. Study Population

**Figure 1:**
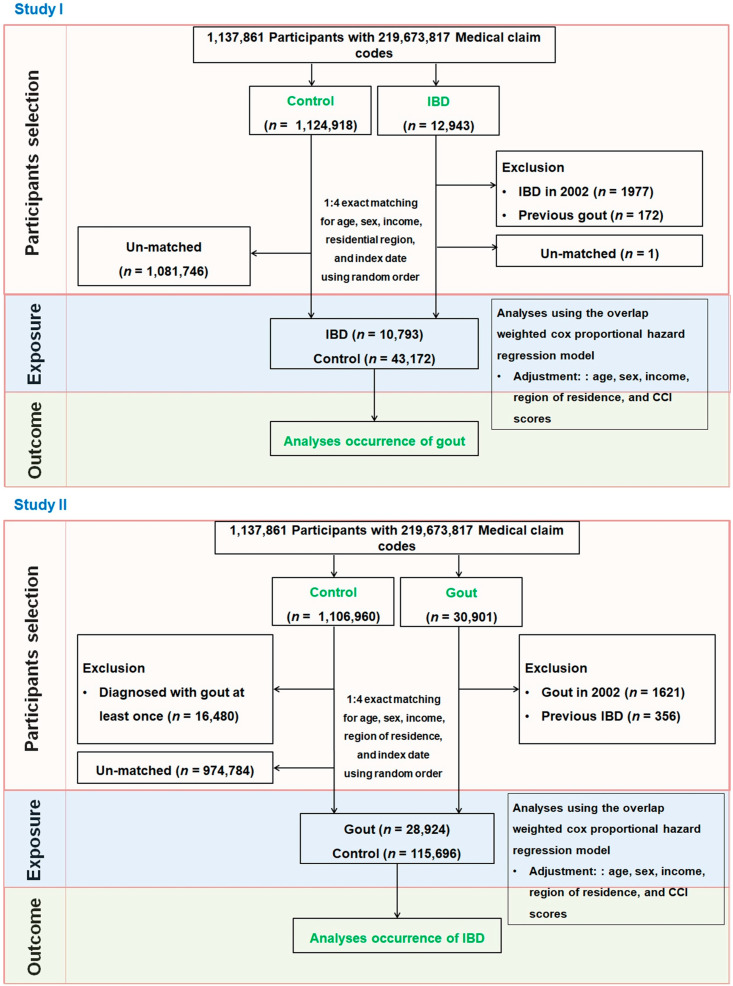
In Study I, 10,793 patients with inflammatory bowel disease (IBD) were matched 1:4 with 43,172 control participants, and the risk of incident gout was evaluated. In Study II, 28,924 patients with gout were matched 1:4 with 115,696 control participants, and the risk of incident IBD was evaluated using overlap-weighted Cox proportional hazards models adjusted for relevant covariates.

We constructed two separate cohorts to evaluate the bidirectional association between IBD and gout (Figure 1).

Study I

A total of 12,943 patients diagnosed with IBD between 2002 and 2019 were identified. We excluded 1977 patients diagnosed in 2002 to establish a new-onset cohort, and 172 patients with a history of gout were also excluded. Stratified 1:4 matching was performed on the basis of sex, age, income level, and residential region to minimize baseline differences and reduce potential confounding. This matching ratio was selected on the basis of prior methodological recommendations, indicating that increasing the number of controls beyond a 4:1 ratio provides little additional statistical efficiency [32]. Non-IBD controls were randomly selected from the general population without an IBD diagnosis during the same period. Controls who died or were diagnosed with gout before the IBD diagnosis date were excluded. Only one IBD patient remained unmatched. The final cohort included 10,793 IBD patients and 43,172 matched controls. The subgroups included 4579 CD patients and 6214 UC patients. Disease-specific analyses were conducted using ICD-10 codes to assess the incidence of gout from the index date until 31 December.

Study II

On the basis of the ICD-10 code M10, 30,901 patients with gout (≥2 outpatient visits) were identified. After excluding those diagnosed in 2002 (*n* = 1621) and those with prior IBD (*n* = 356), 28,924 patients remained. Stratified 1:4 matching was again applied using the same variables. Controls were randomly selected from the population without gout, with those diagnosed with IBD or deceased before the gout diagnosis excluded. No unmatched gout cases were observed. The final cohort consisted of 28,924 gout patients and 115,696 controls. IBD occurrence was tracked via ICD-10 codes until 31 December 2019.

### 2.4. Covariates

Sociodemographic characteristics included age (classified into 18 five-year groups from 0–4 to ≥85 years), sex, income quintiles, and place of residence. Residential location was categorized according to administrative divisions into 16 regional units and subsequently classified as urban or rural. Urban areas were defined as the seven metropolitan cities in Korea, each with a population exceeding one million, whereas rural areas comprised regions with populations below one million [33]. The overall comorbidity burden was measured via the Charlson Comorbidity Index (CCI), which includes 17 medical conditions. The total CCI score ranges from 0 to 29, with higher scores reflecting more severe comorbidity profiles [34].

### 2.5. Statistical Analysis

To minimize confounding and ensure comparability across study groups, we applied overlap weighting on the basis of propensity scores. Propensity scores were estimated via logistic regression models that included all the baseline covariates. Overlap weights were then derived, assigning greater weights to participants with similar probabilities of belonging to either group (gout, IBD, or control), thereby improving comparability and preserving a larger effective sample size compared with conventional matching [35,36]. Balance was assessed via standardized differences, with a value <0.20 considered indicative of acceptable balance [37].

Hazard ratios (HRs) with 95% CIs for the bidirectional relationship between IBD and gout (study I: IBD to gout; study II: gout to IBD) were estimated via overlap-weighted Cox proportional hazards models. The incidence rates per 10,000 person-years and incidence rate differences with 95% CIs were also calculated.

Cumulative incidence rates were estimated via Kaplan–Meier curves and compared via log-rank tests. Subgroup analyses were conducted according to all covariates. All the statistical analyses were performed via SAS version 9.4 (SAS Institute, Cary, NC, USA), with statistical significance defined as *p* < 0.05.

## 3. Results

### 3.1. General Characteristics of the Study Population of Study I and Study II

Table 1 and Table 2 summarize the baseline characteristics of patients with IBD (*n* = 10,793) and gout (*n* = 25,624), respectively, together with their matched control groups (*n* = 43,172 and 115,696). In both studies, the matching process yielded a perfect balance across demographic and socioeconomic factors (age, sex, income level, and residential region), with standardized differences of 0.00. After applying propensity score overlap weighting, the absolute standardized differences for all covariates were reduced to less than 0.20, confirming adequate covariate balance between each disease cohort and its respective control group.

### 3.2. Association Between IBD and Subsequent Risk of Gout (Study I)

During follow-up, patients with IBD had a higher incidence of gout than matched controls (28.40 vs. 24.20 per 10,000 person-years; incidence rate difference [IRD], 4.20; 95% CI, 0.84–7.70). In overlap-weighted Cox models, IBD was associated with a significantly increased risk of incident gout (adjusted HR [aHR], 1.16; 95% CI, 1.04–1.29). When stratified by subtype, CD showed a significant association with gout (aHR, 1.25; 95% CI, 1.05–1.48), whereas UC did not (aHR, 1.11; 95% CI, 0.97–1.27) (Table 3). Kaplan–Meier analyses showed significantly higher cumulative incidence for IBD (log-rank *p* = 0.0147) and CD (log-rank *p* = 0.0277), but not for UC (log-rank *p* = 0.1772) (Supplementary Figure S1).

**Figure 2:**
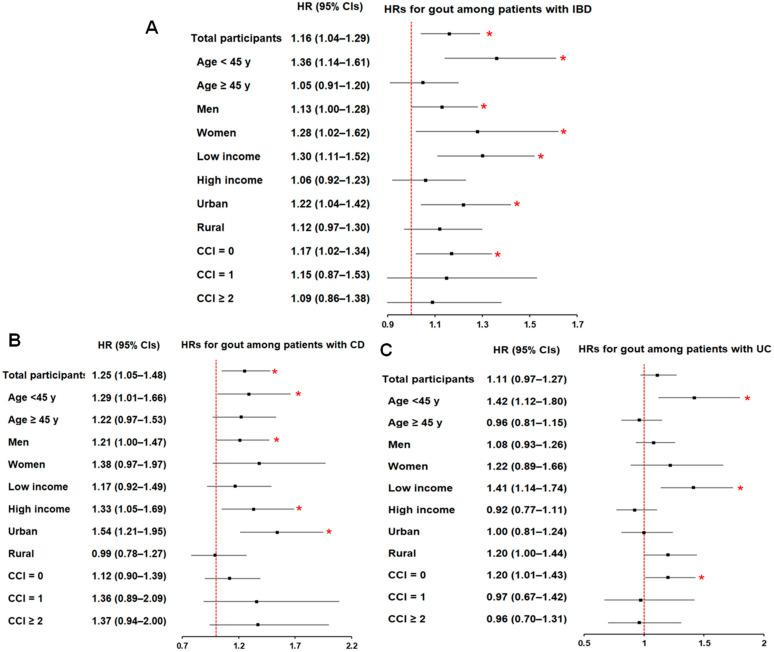
Forest plots of subgroup analyses for the risk of IBD (**A**), CD (**B**), and UC (**C**) in patients with gout compared with matched controls. The red dashed line represents the null value (1.0). * Significance at *p* < 0.05.

Subgroup analyses showed that the association between IBD and gout was more pronounced among younger individuals (<45 years), in both men and women, across income strata, among urban residents, and among individuals with a CCI score of 0 (Supplementary Table S1) (Figure 2A). For CD, significant associations were observed among participants younger than 45 years, men, those with higher income, and urban residents (Supplementary Table S2) (Figure 2B). Although the overall association between UC and gout was not statistically significant, relatively higher risk estimates were observed among younger participants, those with lower income, and those with a CCI score of 0 (Supplementary Table S3) (Figure 2C). Notably, the higher likelihood of gout was significantly associated with IBD participants (and both subtypes, CD and UC) younger than 45 years.

### 3.3. Association Between Gout and Subsequent Risk of IBD (Study II)

In contrast, gout was not significantly associated with the subsequent development of IBD. The incidence of IBD was similar between patients with gout and matched controls (7.51 vs. 7.06 per 10,000 person-years; IRD, 0.45; 95% CI, −0.90 to 1.80), and overlap-weighted analyses showed no significant association (aHR, 1.04; 95% CI, 0.91–1.21). No significant associations were observed for CD (aHR, 1.18; 95% CI, 0.91–1.54) or UC (aHR, 0.98; 95% CI, 0.82–1.18) (Table 4). Kaplan–Meier curves similarly showed no significant differences between groups [IBD (log-rank *p* = 0.5190), CD (*p* = 0.2487), or UC (*p* = 0.9948)] (Supplementary Figure S2).

**Figure 3:**
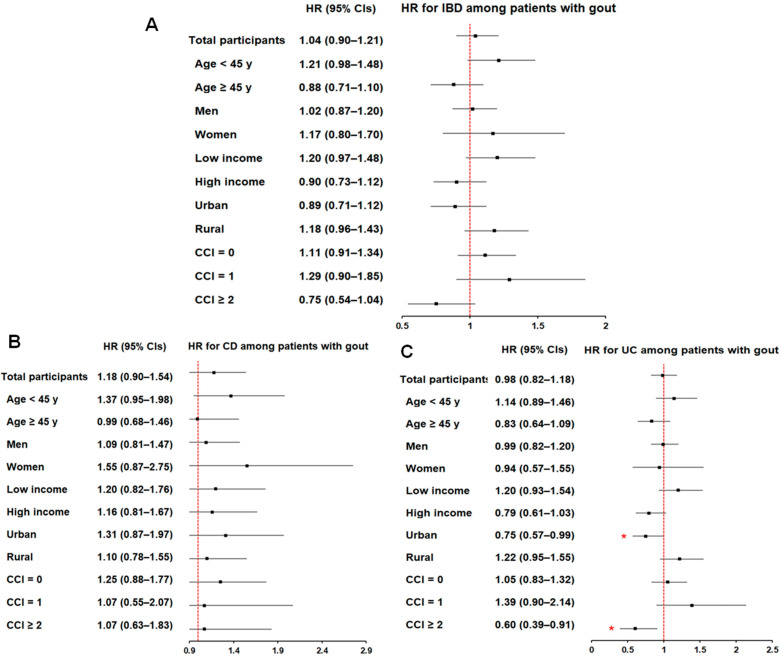
Forest plots of subgroup analyses for the risk of gout in patients with IBD (**A**), CD (**B**), and UC (**C**) compared with matched controls. The red dashed line represents the null value (1.0). * Significance at *p* < 0.05.

Subgroup analyses did not identify consistent or statistically significant associations between gout and the risk of IBD (Supplementary Table S4) (Figure 3A) or its subtypes (Supplementary Table S5–S6) (Figure 3B,C), with few exceptions in UC. Specifically, patients with gout residing in urban areas had a lower risk of UC (aHR, 0.75; 95% CI, 0.57–0.99), as did those with a CCI score of 2 or higher (aHR, 0.60; 95% CI, 0.39–0.91).

## 4. Discussion

To our knowledge, this is among the first nationwide longitudinal cohort studies to evaluate the bidirectional temporal association between IBD and gout. In this nationwide bidirectional cohort study, we observed an asymmetric association between IBD and gout. In Study I, patients with IBD had a higher incidence of gout than matched controls, and IBD was associated with a modest but statistically significant increase in the risk of incident gout after propensity score overlap weighting. The association was more evident for CD, whereas UC did not show a statistically significant overall association. In contrast, Study II did not demonstrate a significant association between gout and subsequent risk of IBD or its subtypes in the overall cohort. Together, these findings may suggest that IBD may confer an increased risk of developing gout, while gout does not appear to meaningfully increase the risk of subsequent IBD at the population level.

In Study I, IBD was associated with a 16% increased risk of developing gout. Although the magnitude of this association was modest, even small relative risk elevations may carry clinical and public health relevance given the chronic and increasingly prevalent nature of both conditions, particularly in the context of population disease burden [38]. A prior epidemiologic study has similarly reported statistically significant but moderate associations between gout and both CD (OR 1.68, 95% CI: 1.60–1.75) and UC (OR 1.38, 95% CI: 1.31–1.44) [24]. The observed relationship may be partly explained by shared inflammatory pathways and alterations in uric acid metabolism and intestinal urate handling, based on prior experimental and translational studies [39,40,41], although the present analysis did not include direct biomarker or functional data to evaluate these pathways. Both IBD and gout involve dysregulated cytokine networks centered on the IL-1β–NLRP3 inflammasome axis, TNF-α, and IL-6, which contribute to systemic inflammatory responses [11,42]. Experimental models of colitis have similarly demonstrated elevations in IL-1β, IL-6, TNF-α, and serum urate levels [39], supporting a biologically plausible link. In addition, anti-TNF therapies commonly used in IBD may indirectly modulate inflammatory pathways relevant to gout [43,44,45,46]. In addition, intestinal dysbiosis characteristic of IBD may influence urate metabolism, as gut microbiota participate in uric acid degradation and host inflammatory regulation [47]. Alterations in gut microbial taxa involved in uric acid metabolism, including *Prevotellaceae*, have been associated with variations in serum urate levels among patients with IBD, suggesting a potential microbiome-mediated pathway [47]. Beyond shared inflammation, altered intestinal urate excretion may further contribute to this association. Approximately one-third of urate is excreted through the gastrointestinal tract [48], a process mediated by transporters such as ABCG2 (ATP-binding cassette transporter superfamily G2), which is expressed in both renal and intestinal tissues and plays a central role in both renal and intestinal excretion of urate [47]. Experimental studies have shown that reduced ABCG2 function and expression in patients with active IBD may impair intestinal urate excretion, thereby contributing to elevated serum uric acid levels and supporting a plausible mechanism for hyperuricemia in this population [41].

When examining disease subtypes, patients with CD showed a significant 25% increased risk of developing gout, whereas UC did not reach statistical significance, indicating that CD may contribute more substantially to the observed association between IBD and gout than UC. The stronger association observed for CD may reflect differences in disease phenotype, extent of intestinal involvement, underlying genetic susceptibility, and uric acid metabolism [3]. Emerging genetic evidence supports a shared biological background between gout and CD. The latest Mendelian randomization analysis using genome-wide association data demonstrated a genetic association between CD and gout risk, revealing the causality of gout on CD but no reverse association between gout and CD risk, particularly in immune regulatory and urate transporter pathways involved in systemic urate homeostasis [49]. A recent cohort study also indicates that elevated serum uric acid levels may be more strongly associated with CD than with UC, further supporting subtype-specific susceptibility [50]. From a pathophysiologic perspective, CD is characterized by transmural inflammation that can involve any segment of the gastrointestinal tract and frequently leads to malabsorption, chronic diarrhea, and surgical resection [3]. Given that approximately one-third of urate excretion occurs through the intestinal mucosa [47], extensive small-bowel involvement in CD may impair extra-renal urate clearance and contribute to hyperuricemia [51]. In contrast, UC is largely confined to the colon and generally preserves small-bowel function, resulting in comparatively limited disruption of intestinal urate handling [3]. CD is also characterized by prominent Th1/Th17 immune activation, which may further interact with urate-driven inflammatory pathways, compared to UC [52]. Cytokines released by these immune cells, including TNF-α, IL-12, IL-23, and IL-17, play pivotal roles in the pathogenesis of CD [52]. The role of Th17 cells is particularly notable, as their production of IL-17 and IL-23 intensifies intestinal inflammatory responses [52]. Consequently, hyperuricemia may worsen CD progression by exacerbating the Th17-mediated immune response [52]. Dehydration and diarrhea, which are more common in CD, may further promote acidic urine and increase renal urate reabsorption, favoring hyperuricemia and uric acid crystallization in CD patients [53]. Supporting this hypothesis, nephrolithiasis is reported to be more prevalent in CD patients than in UC patients, and the risk of gout has been reported to increase more than twofold after ileal resection (OR 2.34) in CD patients [24,54]. A hospital-based study similarly demonstrated higher uric acid–to–creatinine ratios in patients with CD but not UC [23]. However, these proposed mechanisms remain speculative and warrant further investigation.

Additionally, intestinal dysbiosis observed in CD has been reported to influence uric acid metabolism and renal excretion [20,21,23]. Dehydration and diarrhea, which are more common in CD, may further promote acidic urine and increase renal urate reabsorption, favoring hyperuricemia and uric acid crystallization [53]. Supporting this hypothesis, nephrolithiasis is reported to be more prevalent in CD patients than in UC patients, and the risk of gout has been reported to increase more than twofold after ileal resection (OR 2.34) [24,54]. A hospital-based study demonstrated that the uric acid–to–creatinine ratios were significantly increased in patients with CD, whereas no such association was observed in patients with UC [23]. Furthermore, these discrepancies may reflect differences in study design [24]. Our cohort excluded individuals with preexisting gout to focus on incident risk, whereas the prior large database study reported prevalence estimates without such exclusions [24]. However, these are only possible mechanisms, and further studies are needed to clarify the specific mechanisms.

Interestingly, subgroup analyses (Study I) demonstrated that the association between IBD patients and the likelihood of developing gout was more evident among younger individuals (<45 years), men, urban residents, and those without comorbidities. A notable finding was that all IBD and its subtypes (CD and UC) were significantly associated with gout among individuals younger than 45 years. The stronger association with gout likelihood observed in younger IBD patients without significant comorbidities may suggest a genetically mediated or inflammation-dominant pathway, rather than one driven by cumulative metabolic risk [7,39]. Unlike the female predominance commonly reported in Western populations with CD [1], a pronounced male predominance is observed in both CD and UC in Korea, potentially reflecting regional and epidemiological differences in disease expression [23,24,39]. The incidence of IBD in Korea has been rising steadily, largely attributed to the increasing adoption of a Westernized lifestyle and diet [55,56]. Urban–rural differences are evident, with Seoul exhibiting nearly twice the incidence observed in rural Jeollanam-do [9]. Similarly, regional variations in China correlate with population density [7], suggesting that socioeconomic and lifestyle factors may play a significant role in gout development among patients with IBD.

Conversely, in our reverse-direction analysis (Study II), patients with gout did not have a significantly increased risk of subsequent IBD. The incidence of IBD did not differ meaningfully between the gout and control groups, and adjusted analyses yielded an aHR of 1.04 (95% CI, 0.91–1.21). Subtype analyses for CD and UC yielded similar null results. This null finding is consistent with recent Mendelian randomization evidence demonstrating no causal relationship between gout and IBD [39]. Specifically, genetically elevated serum urate was not associated with CD or overall IBD [39]. Furthermore, animal models of colitis induced by dextran sulfate sodium exhibit increased serum urate levels along with elevated IL-6, IL-1β, and TNF-α levels, suggesting that hyperuricemia may reflect a downstream effect of intestinal inflammation rather than a causal driver [39]. These findings suggest that hyperuricemia and gout may not be sufficient to trigger chronic intestinal inflammation leading to IBD onset.

In Study II, the few inverse subgroup associations observed for UC (e.g., among urban residents or those with higher comorbidity burden) should be interpreted cautiously. Given multiple subgroup comparisons, these findings could reflect chance, competing risks, differential healthcare utilization, or diagnostic coding patterns rather than a true protective effect. In particular, individuals with higher comorbidity burden may have competing mortality. Nevertheless, an animal study by Rahimian et al. demonstrated that uric acid mediated the protective effects of inosine against colitis [57], and a Mendelian randomization study described that genetically predicted IBD was inversely correlated with serum urate levels (OR 0.97), consistent with our result [39], warranting further study.

This study has several limitations. First, as with all claims-based analyses, residual confounding cfannot be excluded because detailed clinical and laboratory information—including serum urate levels, dietary factors, medication exposure, and biomarker or genetic data—was not available. Consequently, these variables could not be incorporated into the analyses, limiting adjustment for potential confounders and precluding direct evaluation of the biological mechanisms underlying the observed associations. Second, disease definitions rely on diagnostic codes, increasing the possibility of misclassification. Third, because the cohort was derived from a single national database, generalizability to other populations may be limited.

Nevertheless, this study also has important strengths. First, we leveraged a large nationwide cohort with long-term follow-up, enabling evaluation of relatively uncommon incident outcomes and supporting subgroup analyses. Second, our bidirectional matched cohort design addressed temporal directionality and reduced concern for reverse causation compared with cross-sectional studies. Prior epidemiologic studies have primarily examined a single direction—typically the risk of gout among patients with IBD—or relied on cross-sectional or prevalence-based comparisons that are more vulnerable to reverse causation and confounding. In contrast, our complementary two-cohort framework enabled evaluation of temporal directionality (from IBD to gout and from gout to IBD) within the same population-based data source, thereby providing a more reliable assessment of whether these conditions are linked asymmetrically or bidirectionally. Furthermore, by focusing on incident cases and excluding individuals with preexisting disease, we could better establish temporal relationships and reduce reverse causality. Third, our two-step confounding control strategy—exact matching followed by propensity score overlap weighting—achieved strong covariate balance and enhanced internal validity. Subtype-specific analyses distinguished CD from UC, while comprehensive matching with Cox proportional hazards modeling enhanced the validity of time–event comparisons and minimized selection bias. Finally, concordance between incidence rate comparisons, HR estimates, and Kaplan–Meier curves may support the reliability of the observed patterns.

## 5. Conclusions

In our bidirectional results, IBD was associated with a modest but statistically significant increase in the risk of developing gout, whereas gout did not significantly influence the subsequent risk of IBD. These findings may support a subtype-specific and directionally asymmetric relationship, wherein IBD (especially CD) may increase the risk of gout, whereas gout itself appears unlikely to play a causal role in the development of IBD. From a clinical perspective, clinicians caring for patients with IBD, especially younger individuals and those with CD, should remain attentive to gout-related symptoms to facilitate timely recognition and management of this potential extraintestinal comorbidity.

## Figures and Tables

**Table 1:** Baseline demographic and clinical characteristics of participants with IBD and matched controls in Study I before and after propensity score overlap weighting.

Characteristics	Before PS Overlap Weighting Adjustment			After PS Overlap Weighting Adjustment		
	IBD	Control	Standardized Difference	IBD	Control	Standardized Difference
Age (%)			0.00			0.00
0–4	355 (3.29)	1420 (3.29)		284 (3.29)	284 (3.29)	
5–9	306 (2.84)	1224 (2.84)		245 (2.84)	245 (2.84)	
10–14	361 (3.34)	1444 (3.34)		289 (3.35)	289 (3.35)	
15–19	550 (5.10)	2200 (5.10)		439 (5.10)	439 (5.10)	
20–24	651 (6.03)	2604 (6.03)		520 (6.04)	520 (6.04)	
25–29	732 (6.78)	2928 (6.78)		585 (6.79)	585 (6.79)	
30–34	798 (7.39)	3192 (7.39)		638 (7.40)	638 (7.40)	
35–39	760 (7.04)	3040 (7.04)		607 (7.04)	607 (7.04)	
40–44	881 (8.16)	3524 (8.16)		704 (8.17)	704 (8.17)	
45–49	911 (8.44)	3644 (8.44)		728 (8.44)	728 (8.44)	
50–54	975 (9.03)	3900 (9.03)		781 (9.06)	781 (9.06)	
55–59	857 (7.94)	3428 (7.94)		684 (7.93)	684 (7.93)	
60–64	753 (6.98)	3012 (6.98)		601 (6.97)	601 (6.97)	
65–69	742 (6.87)	2968 (6.87)		592 (6.87)	592 (6.87)	
70–74	556 (5.15)	2224 (5.15)		442 (5.13)	442 (5.13)	
75–79	340 (3.15)	1360 (3.15)		271 (3.14)	271 (3.14)	
80–84	180 (1.67)	720 (1.67)		144 (1.67)	144 (1.67)	
85+	85 (0.79)	340 (0.79)		68 (0.78)	68 (0.78)	
Sex (%)			0.00			0.00
Male	5601 (51.89)	22,404 (51.89)		4473 (51.90)	4473 (51.90)	
Female	5192 (48.11)	20,768 (48.11)		4146 (48.10)	4146 (48.10)	
Income (%)			0.00			0.00
1 (lowest)	1809 (16.76)	7236 (16.76)		1443 (16.74)	1443 (16.74)	
2	1524 (14.12)	6096 (14.12)		1218 (14.13)	1218 (14.13)	
3	1957 (18.13)	7828 (18.13)		1562 (18.13)	1563 (18.13)	
4	2492 (23.09)	9968 (23.09)		1991 (23.10)	1991 (23.10)	
5 (highest)	3011 (27.90)	12,044 (27.90)		2405 (27.90)	2405 (27.90)	
Region of residence (%)			0.00			0.00
Urban	4820 (44.66)	19,280 (44.66)		3851 (44.68)	3851 (44.68)	
Rural	5973 (55.34)	23,892 (55.34)		4768 (55.32)	4768 (55.32)	
CCI score (Mean, SD)	0.71 (1.48)	0.58 (1.34)	0.1	0.68 (1.28)	0.68 (0.67)	0.00
Gout (*n*, %)	290 (2.69)	987 (2.29)	0.03	231 (2.68)	199 (2.31)	0.02

Abbreviations: IBD, inflammatory bowel disease; CCI, Charlson Comorbidity Index; PS, propensity score; SD, standard deviation. Covariate balance between groups was assessed using standardized differences, with absolute standardized differences <0.20 indicating adequate balance.

**Table 2:** Baseline demographic and clinical characteristics of participants with gout and matched controls in Study II before and after propensity score overlap weighting.

Characteristics	Before PS Overlap Weighting Adjustment			After PS Overlap Weighting Adjustment		
	Gout	Control	Standardized Difference	Gout	Control	Standardized Difference
Age (%)			0.00			0.00
0–4	1 (0.00)	4 (0.00)		1 (0.00)	1 (0.00)	
5–9	7 (0.02)	28 (0.02)		6 (0.02)	6 (0.02)	
10–14	53 (0.18)	212 (0.18)		42 (0.18)	42 (0.18)	
15–19	262 (0.91)	1048 (0.91)		209 (0.91)	209 (0.91)	
20–24	747 (2.58)	2988 (2.58)		597 (2.59)	597 (2.59)	
25–29	1440 (4.98)	5760 (4.98)		1151 (4.99)	1151 (4.99)	
30–34	2164 (7.48)	8656 (7.48)		1728 (7.49)	1728 (7.49)	
35–39	2744 (9.49)	10,976 (9.49)		2191 (9.50)	2191 (9.50)	
40–44	3005 (10.39)	12,020 (10.39)		2399 (10.40)	2399 (10.40)	
45–49	3310 (11.44)	13,240 (11.44)		2643 (11.46)	2643 (11.46)	
50–54	3396 (11.74)	13,584 (11.74)		2709 (11.74)	2709 (11.74)	
55–59	3049 (10.54)	12,196 (10.54)		2431 (10.54)	2431 (10.54)	
60–64	2683 (9.28)	10,732 (9.28)		2138 (9.27)	2138 (9.27)	
65–69	2154 (7.45)	8616 (7.45)		1713 (7.43)	1714 (7.43)	
70–74	1748 (6.04)	6992 (6.04)		1393 (6.04)	1393 (6.04)	
75–79	1178 (4.07)	4712 (4.07)		937 (4.06)	937 (4.06)	
80–84	639 (2.21)	2556 (2.21)		508 (2.20)	508 (2.20)	
85+	344 (1.19)	1376 (1.19)		273 (1.18)	273 (1.18)	
Sex (%)			0.00			0.00
Male	23,042 (79.66)	92,168 (79.66)		18,388 (79.71)	18,389 (79.71)	
Female	5882 (20.34)	23,528 (20.34)		4680 (20.29)	4680 (20.29)	
Income (%)			0.00			0.00
1 (lowest)	5094 (17.61)	20,376 (17.61)		4054 (17.57)	4054 (17.58)	
2	4097 (14.16)	16,388 (14.16)		3269 (14.17)	3269 (14.17)	
3	5223 (18.06)	20,892 (18.06)		4168 (18.07)	4168 (18.07)	
4	6427 (22.22)	25,708 (22.22)		5129 (22.23)	5129 (22.23)	
5 (highest)	8083 (27.95)	32,332 (27.95)		6449 (27.95)	6449 (27.95)	
Region of residence (%)			0.00			0.00
Urban	12,791 (44.22)	51,164 (44.22)		10,204 (44.23)	10,204 (44.23)	
Rural	16,133 (55.78)	64,532 (55.78)		12,864 (55.77)	12,865 (55.77)	
CCI score (Mean, SD)	0.84 (1.59)	0.65 (1.41)	0.13	0.79 (1.36)	0.79 (0.73)	0.00
IBD (*n*, %)	142 (0.49)	535 (0.46)	0.00	113 (0.49)	109 (0.47)	0.00
CD (*n*, %)	48 (0.17)	159 (0.14)	0.01	38 (0.16)	33 (0.14)	0.01
UC (*n*, %)	94 (0.32)	376 (0.32)	0.00	75 (0.32)	77 (0.33)	0.00

Abbreviations: IBD, inflammatory bowel disease; CD, Crohn’s disease; UC, ulcerative colitis; CCI, Charlson comorbidity index; PS, propensity score; SD, standard deviation. Covariate balance between groups was assessed using standardized differences, with absolute standardized differences <0.20 indicating adequate balance.

**Table 3:** Incidence rates and hazard ratios for gout in patients with IBD and matched controls.

Outcome	Exposure	N of Event/ N of Total (%)	IR per 10,000 (PY)	IRD (95% CI)	Hazard Ratios for Gout (95% Confidence Intervals)			
					Crude	*p*	Adjusted Model with OW †	*p*
Gout	IBD	290/10,793 (2.69)	28.40	4.20 (0.84–7.70)	1.18 (1.03–1.34)	0.015 *	1.16 (1.04–1.29)	0.006 *
	Non-IBD	987/43,172 (2.29)	24.20		1 (ref)		1 (ref)	
	CD	121/4579 (2.64)	25.90	5.30 (0.59–10.04)	1.26 (1.02–1.54)	0.028 *	1.25 (1.05–1.48)	0.010 *
	Non-CD	387/18,316 (2.11)	20.60		1 (ref)		1 (ref)	
	UC	169/6214 (2.72)	30.50	3.30 (−1.54–8.29)	1.12 (0.95–1.33)	0.177	1.11 (0.97–1.27)	0.145
	Non-UC	600/24,856 (2.41)	27.20		1 (ref)		1 (ref)	

Abbreviations: IR, incidence rate; IRD, incidence rate difference; PY, person-year; OW, overlap weighting; IBD, inflammatory bowel disease; CD, Crohn’s disease; UC, ulcerative colitis. * Significance at *p* < 0.05. † Adjusted for age, sex, income, region of residence, and CCI scores. Hazard ratios were estimated using Cox proportional hazards regression models. In study I, CD and UC represent subgroups of the overall IBD cohort; therefore, the number of participants differed across analyses.

**Table 4:** Incidence rates and hazard ratios for IBD in patients with gout and matched controls.

Outcome	Exposure	N of Event/ N of Total (%)	IR per 10,000 (PY)	IRD (95% CI)	Hazard Ratio (95% Confidence Intervals)			
					Crude	*p*	Adjusted Model with OW †	*p*
IBD	Gout	142/28,924 (0.49)	7.51	0.45 (−0.90–1.80)	1.06 (0.88–1.28)	0.519	1.04 (0.9–1.21)	0.580
	Non-gout	535/115,696 (0.46)	7.06		1 (ref)		1 (ref)	
CD	Gout	48/28,924 (0.17)	2.53	0.44 (−0.30–1.18)	1.21 (0.88–1.67)	0.249	1.18 (0.9–1.54)	0.224
	Non-gout	159/115,696 (0.14)	2.09		1 (ref)		1 (ref)	
UC	Gout	94/28,924 (0.32)	4.96	0.01 (−1.11–1.13)	1.00 (0.80–1.25)	1.00	0.98 (0.82–1.18)	0.866
	Non-gout	376/115,696 (0.32)	4.95		1 (ref)		1 (ref)	

Abbreviations: IR, incidence rate; IRD, incidence rate difference; PY, person-year; OW, overlap weighting; IBD, inflammatory bowel disease. † Adjusted for age, sex, income, region of residence, and CCI scores. Hazard ratios were estimated using Cox proportional hazards regression models.

## Data Availability

All data are available from the database of the National Health Insurance Sharing Service (NHISS) https://nhiss.nhis.or.kr/ (accessed on 23 January 2026). The NHISS allows access to all of these data for any researcher who agrees to follow the research ethics for a processing charge. If you want to access the data of this article, you can download it from the website after promising to follow the research ethics.

## Supplementary Materials

The following supporting information can be downloaded at: https://www.mdpi.com/article/10.3390/biomedicines14030613/s1. Table S1: Crude and adjusted hazard ratios (95% confidence interval) of IBD for gout with subgroup analyses according to age, sex, income, region, and CCI scores. Table S2: Crude and adjusted hazard ratios (95% confidence interval) of CD for gout with subgroup analyses according to age, sex, income, region, and CCI scores. Table S3: Crude and adjusted hazard ratios (95% confidence interval) of UC for gout with subgroup analyses according to age, sex, income, region, and CCI scores. Table S4: Crude and adjusted hazard ratios (95% confidence interval) of gout for IBD with subgroup analyses according to age, sex, income, region, and CCI scores. Table S5: Crude and adjusted hazard ratios (95% confidence interval) of gout for CD with subgroup analyses according to age, sex, income, region, and CCI scores. Table S6: Crude and adjusted hazard ratios (95% confidence interval) of gout for UC with subgroup analyses according to age, sex, income, region, and CCI scores. Figure S1: Kaplan–Meier analyses showed significantly higher cumulative incidence for IBD (log-rank *p* = 0.0147) and CD (log-rank *p* = 0.0277), but not for UC (log-rank *p* = 0.1772). Figure S2: Kaplan–Meier curves similarly showed no significant differences between groups [IBD (log-rank *p* = 0.5190), CD (*p* = 0.2487), or UC (*p* = 0.9948)].

## Abbreviations

The following abbreviations are used in this manuscript:

IBD	Inflammatory bowel disease
CD	Crohn’s disease
UC	Ulcerative colitis
PS	Propensity score
OW	Overlap weighting
CCI	Charlson Comorbidity Index
HR	Hazard ratio
