# Supplementary material for: A Bidirectional Analysis of Inflammatory Bowel Disease and Gout: Epidemiologic Evidence of a Stronger Association in Crohn’s Disease from a Nationwide Cohort Study in South Korea
Source: Biomedicines. 2026 Mar 9;14(3):613. doi: 10.3390/biomedicines14030613 (PMC13024287; doi:10.3390/biomedicines14030613)

Supplementary Table S1. Crude and adjusted hazard ratios (95% confidence interval) of IBD for gout with subgroup analyses according to age, sex, income, region, and CCI scores

	N of event / N of total (%)	Follow-up duration (PY)	IR per 10000 (PY)	IRD (95% CI)	Hazard ratios for gout			
					Crude	P-value	Adjusted model with OW †	P-value
Total participants (n = 53,965)								
IBD	290 / 10,793 (2.69)	101,985	28.40	4.20 (0.84 to 7.70)	1.18 (1.03-1.34)	0.015*	1.16 (1.04-1.29)	0.006*
Control	987 / 43,172 (2.29)	408,472	24.20		1		1	
Age < 45 years old (n = 26,970)								
IBD	123 / 5,394 (2.28)	55,925	22.00	5.90 (2.05 to 9.75)	1.37 (1.11-1.68)	0.003*	1.36 (1.14-1.61)	<0.001*
Control	361 / 21,576 (1.67)	224,364	16.10		1		1	
Age ≥ 45 years old (n = 26,995)								
IBD	167 / 5,399 (3.09)	46,060	36.30	2.30 (-3.74 to 8.25)	1.07 (0.90-1.27)	0.458	1.05 (0.91-1.20)	0.491
Control	626 / 21,596 (2.90)	184,108	34.00		1		1	
Male (n = 28,005)								
IBD	225 / 5,601 (4.02)	51,011	44.10	5.50 (-0.60 to 11.63)	1.14 (0.99-1.33)	0.077	1.13 (1.00-1.28)	0.046*
Control	786 / 22,404 (3.51)	203,674	38.60		1		1	
Female (n = 25,960)								
IBD	65 / 5,192 (1.25)	50,974	12.80	2.99 (-0.19 to 6.07)	1.30 (0.98-1.72)	0.066	1.28 (1.02-1.62)	0.035*
Control	201 / 20,768 (0.97)	204,798	9.81		1		1	
Low income group (n = 26,450)								
IBD	140 / 5,290 (2.65)	49,466	28.30	6.70 (2.02 to 11.45)	1.31 (1.08-1.59)	0.005*	1.30 (1.11-1.52)	0.001*
Control	428 / 21,160 (2.02)	198,456	21.60		1		1	
High income group (n = 27,515)								
IBD	150 / 5,503 (2.73)	52,519	28.60	2.00 (-3.03 to 6.91)	1.07 (0.90-1.28)	0.445	1.06 (0.92-1.23)	0.418
Control	559 / 22,012 (2.54)	210,016	26.60		1		1	
Urban resident (n = 24,100)								
IBD	138 / 4,820 (2.86)	45,883	30.10	5.60 (0.40 to 10.75)	1.23 (1.01-1.49)	0.035*	1.22 (1.04-1.42)	0.015*

Control	450 / 19,280 (2.33)	183,647	24.50		1		1	
Rural resident (n = 29,865)								
IBD	152 / 5,973 (2.54)	56,102	27.10	3.20 (-1.37 to 7.79)	1.13 (0.95-1.36)	0.17	1.12 (0.97-1.30)	0.128
Control	537 / 23,892 (2.25)	224,825	23.90		1		1	
CCI scores = 0 (n = 39,781)								
IBD	173 / 7,478 (2.31)	71,807	24.10	3.50 (-0.30 to 7.19)	1.17 (0.99-1.38)	0.073	1.17 (1.02-1.34)	0.022*
Control	640 / 32,303 (1.98)	309,961	20.60		1		1	
CCI scores = 1 (n = 6,644)								
IBD	51 / 1,554 (3.28)	15,011	34.00	3.80 (-6.44 to 14.02)	1.13 (0.82-1.55)	0.459	1.15 (0.87-1.53)	0.315
Control	144 / 5,090 (2.83)	47,701	30.20		1		1	
CCI scores ≥ 2 (n = 7,540)								
IBD	66 / 1,761 (3.75)	15,167	43.50	3.50 (-8.02 to 15.14)	1.09 (0.82-1.43)	0.565	1.09 (0.86-1.38)	0.498
Control	203 / 5,779 (3.51)	50,810	40.00		1		1	

Abbreviation: IR, incidence rate; IRD, incidence rate difference; PY. person-year; OW, overlap weighting; IBD, Inflammatory bowel disease;

* Significance at P < 0.05

† Adjusted for age, sex, income, and region of residence, and CCI scores

Supplementary Table S2. Crude and adjusted hazard ratios (95% confidence interval) of CD for gout with subgroup analyses according to age, sex, income, region, and CCI scores

	N of event / N of total (%)	Follow-up duration (PY)	IR per 10000 (PY)	IRD (95% CI)	Hazard ratios for gout			
					Crude	P-value	Adjusted model with OW †	P-value
Total participants (n = 22,895)								
CD	121 / 4,579 (2.64)	46,637	25.90	5.30 (0.59 to 10.04)	1.26 (1.02-1.54)	0.028*	1.25 (1.05-1.48)	0.01*
Control	387 / 18,316 (2.11)	187,571	20.60		1		1	
Age < 45 years old (n = 13,740)								
CD	56 / 2,748 (2.04)	29,413	19.00	4.20 (-0.77 to 9.32)	1.29 (0.95-1.74)	0.097	1.29 (1.01-1.66)	0.044*
Control	174 / 10,992 (1.58)	117,864	14.80		1		1	
Age ≥ 45 years old (n = 9,155)								
CD	65 / 1,831 (3.55)	17,224	37.70	7.10 (-2.25 to 16.61)	1.23 (0.93-1.63)	0.138	1.22 (0.97-1.53)	0.093
Control	213 / 7,324 (2.91)	69,707	30.60		1		1	
Male (n = 11,785)								
CD	92 / 2,357 (3.90)	23,337	39.40	7.20 (-1.10 to 15.54)	1.22 (0.97-1.54)	0.091	1.21 (1.00-1.47)	0.048*
Control	301 / 9,428 (3.19)	93,470	32.20		1		1	
Female (n = 11,110)								
CD	29 / 2,222 (1.31)	23,300	12.40	3.26 (-1.18 to 7.80)	1.36 (0.89-2.08)	0.15	1.38 (0.97-1.97)	0.071
Control	86 / 8,888 (0.97)	94,101	9.14		1		1	
Low income group (n = 11,645)								
CD	57 / 2,329 (2.45)	23,759	24.00	3.70 (-2.83 to 10.21)	1.18 (0.88-1.59)	0.267	1.17 (0.92-1.49)	0.209
Control	194 / 9,316 (2.08)	95,555	20.30		1		1	
High income group (n = 11,250)								
CD	64 / 2,250 (2.84)	22,878	28.00	7.00 (0.15 to 13.85)	1.33 (1.00-1.77)	0.047*	1.33 (1.05-1.69)	0.017*
Control	193 / 9,000 (2.14)	92,016	21.00		1		1	
Urban resident (n = 10,500)								
CD	70 / 2,100 (3.33)	22,165	31.60	11.10 (4.09 to 18.10)	1.54 (1.17-2.03)	0.002*	1.54 (1.21-1.95)	<0.001*

Control	183 / 8,400 (2.18)	89,323	20.50		1		1	
Rural resident (n = 12,395)								
CD	51 / 2,479 (2.06)	24,472	20.80	0.00 (-6.31 to 6.46)	1.00 (0.74-1.36)	0.983	0.99 (0.78-1.27)	0.968
Control	204 / 9,916 (2.06)	98,248	20.80		1		1	
CCI scores = 0 (n = 17,418)								
CD	66 / 3,287 (2.01)	34,322	19.20	1.90 (-2.96 to 6.91)	1.11 (0.85-1.46)	0.443	1.12 (0.90-1.39)	0.31
Control	252 / 14,131 (1.78)	146,052	17.30		1		1	
CCI scores = 1 (n = 2,647)								
CD	26 / 635 (4.09)	6,510	39.90	11.60 (-3.91 to 27.19)	1.41 (0.89-2.24)	0.143	1.36 (0.89-2.09)	0.154
Control	58 / 2,012 (2.88)	20,497	28.30		1		1	
CCI scores ≥ 2 (n = 2,830)								
CD	29 / 657 (4.41)	5,805	50.00	13.40 (-4.94 to 31.60)	1.35 (0.88-2.07)	0.171	1.37 (0.94-2.00)	0.101
Control	77 / 2,173 (3.54)	21,022	36.60		1		1	

Abbreviation: IR, incidence rate; IRD, incidence rate difference; PY. person-year; OW, overlap weighting; CD, Crohn's disease;

* Significance at P < 0.05

† Adjusted for age, sex, income, and region of residence, and CCI scores

Supplementary Table S3. Crude and adjusted hazard ratios (95% confidence interval) of UC for gout with subgroup analyses according to age, sex, income, region, and CCI scores

	N of event / N of total (%)	Follow-up duration (PY)	IR per 10000 (PY)	IRD (95% CI)	Hazard ratios for gout			
					Crude	P-value	Adjusted model with OW †	P-value
Total participants (n = 31,070)								
UC	169 / 6,214 (2.72)	55,348	30.50	3.30 (-1.54 to 8.29)	1.12 (0.95-1.33)	0.177	1.11 (0.97-1.27)	0.145
Control	600 / 24,856 (2.41)	220,901	27.20		1		1	
Age < 45 years old (n = 13,230)								
UC	67 / 2,646 (2.53)	26,512	25.30	7.70 (1.83 to 13.59)	1.44 (1.09-1.90)	0.01*	1.42 (1.12-1.80)	0.003*
Control	187 / 10,584 (1.77)	106,500	17.60		1		1	
Age ≥ 45 years old (n = 17,840)								
UC	102 / 3,568 (2.86)	28,836	35.40	-0.70 (-8.47 to 7.02)	0.98 (0.79-1.22)	0.865	0.96 (0.81-1.15)	0.686
Control	413 / 14,272 (2.89)	114,401	36.10		1		1	
Male (n = 16,220)								
UC	133 / 3,244 (4.10)	27,674	48.10	4.10 (-4.77 to 12.87)	1.09 (0.90-1.32)	0.365	1.08 (0.93-1.26)	0.327
Control	485 / 12,976 (3.74)	110,204	44.00		1		1	
Female (n = 14,850)								
UC	36 / 2,970 (1.21)	27,674	13.00	2.60 (-1.73 to 6.97)	1.25 (0.86-1.82)	0.239	1.22 (0.89-1.66)	0.217
Control	115 / 11,880 (0.97)	110,697	10.40		1		1	
Low income group (n = 14,805)								
UC	83 / 2,961 (2.80)	25,707	32.30	9.60 (2.76 to 16.33)	1.42 (1.11-1.83)	0.006*	1.41 (1.14-1.74)	0.001*
Control	234 / 11,844 (1.98)	102,901	22.70		1		1	
High income group (n = 16,265)								
UC	86 / 3,253 (2.64)	29,641	29.00	-2.00 (-9.05 to 5.04)	0.94 (0.74-1.18)	0.581	0.92 (0.77-1.11)	0.4
Control	366 / 13,012 (2.81)	118,000	31.00		1		1	
Urban resident (n = 13,600)								
UC	68 / 2,720 (2.50)	23,718	28.70	0.40 (-7.22 to 7.95)	1.01 (0.78-1.32)	0.918	1.00 (0.81-1.24)	0.968

Control	267 / 10,880 (2.45)	94,324	28.30		1		1	
Rural resident (n = 17,470)								
UC	101 / 3,494 (2.89)	31,630	31.90	5.60 (-0.83 to 12.08)	1.21 (0.97-1.52)	0.088	1.20 (1.00-1.44)	0.055
Control	333 / 13,976 (2.38)	126,577	26.30		1		1	
CCI scores = 0 (n = 22,363)								
UC	107 / 4,191 (2.55)	37,485	28.50	4.80 (-0.69 to 10.44)	1.21 (0.97-1.49)	0.087	1.20 (1.01-1.43)	0.036*
Control	388 / 18,172 (2.14)	163,909	23.70		1		1	
CCI scores = 1 (n = 3,997)								
UC	25 / 919 (2.72)	8,501	29.40	-2.20 (-15.78 to 11.37)	0.93 (0.60-1.46)	0.767	0.97 (0.67-1.42)	0.885
Control	86 / 3,078 (2.79)	27,204	31.60		1		1	
CCI scores ≥ 2 (n = 4,710)								
UC	37 / 1,104 (3.35)	9,362	39.50	-2.80 (-17.76 to 12.21)	0.94 (0.65-1.36)	0.747	0.96 (0.70-1.31)	0.798
Control	126 / 3,606 (3.49)	29,788	42.30		1		1	

Abbreviation: IR, incidence rate; IRD, incidence rate difference; PY. person-year; OW, overlap weighting; UC, Ulcerative colitis;
* Significance at P < 0.05
† Adjusted for age, sex, income, and region of residence, and CCI scores

Supplementary Table S4. Crude and adjusted hazard ratios (95% confidence interval) of gout for IBD with subgroup analyses according to age, sex, income, region, and CCI scores

	N of event / N of total (%)	Follow-up duration (PY)	IR per 10000 (PY)	IRD (95% CI)	Hazard ratios for IBD			
					Crude	P-value	Adjusted model with OW †	P-value
Total participants (n = 144,620)								
Gout	142 / 28,924 (0.49)	189,192	7.51	0.45 (-0.90 to 1.80)	1.06 (0.88-1.28)	0.519	1.04 (0.9-1.21)	0.58
Control	535 / 115,696 (0.46)	758,120	7.06		1		1	
Age < 45 years old (n = 85,645)								
Gout	81 / 17,129 (0.47)	118,684	6.82	1.22 (-0.31 to 2.76)	1.22 (0.95-1.56)	0.119	1.21 (0.98-1.48)	0.07
Control	266 / 68,516 (0.39)	475,028	5.60		1		1	
Age ≥ 45 years old (n = 58,975)								
Gout	61 / 11,795 (0.52)	70,508	8.65	-0.85 (-3.37 to 1.67)	0.91 (0.69-1.20)	0.498	0.88 (0.71-1.10)	0.26
Control	269 / 47,180 (0.57)	283,092	9.50		1		1	
Male (n = 115,210)								
Gout	118 / 23,042 (0.51)	151,303	7.80	0.25 (-1.31 to 1.80)	1.03 (0.84-1.26)	0.755	1.02 (0.87-1.20)	0.833
Control	456 / 92,168 (0.49)	603,841	7.55		1		1	
Female (n = 29,410)								
Gout	24 / 5,882 (0.41)	37,889	6.33	1.21 (-1.39 to 3.82)	1.23 (0.78-1.95)	0.367	1.17 (0.80-1.70)	0.411
Control	79 / 23,528 (0.34)	154,279	5.12		1		1	
Low income group (n = 72,070)								
Gout	77 / 14,414 (0.53)	92,348	8.34	1.60 (-0.32 to 3.51)	1.24 (0.96-1.60)	0.105	1.20 (0.97-1.48)	0.092
Control	250 / 57,656 (0.43)	370,854	6.74		1		1	
High income group (n = 72,550)								
Gout	65 / 14,510 (0.45)	96,844	6.71	-0.65 (-2.54 to 1.25)	0.91 (0.70-1.19)	0.5	0.90 (0.73-1.12)	0.354
Control	285 / 58,040 (0.49)	387,266	7.36		1		1	
Urban resident (n = 63,955)								
Gout	57 / 12,791 (0.45)	85,127	6.70	-0.75 (-2.78 to 1.28)	0.90 (0.67-1.20)	0.466	0.89 (0.71-1.12)	0.318

Control	254 / 51,164 (0.50)	340,980	7.45		1		1	
Rural resident (n = 80,665)								
Gout	85 / 16,133 (0.53)	104,065	8.17	1.43 (-0.37 to 3.23)	1.21 (0.95-1.54)	0.122	1.18 (0.96-1.43)	0.109
Control	281 / 64,532 (0.44)	417,140	6.74		1		1	
CCI scores = 0 (n = 102,517)								
Gout	81 / 18,985 (0.43)	120,990	6.69	0.67 (-0.87 to 2.22)	1.11 (0.87-1.42)	0.4	1.11 (0.91-1.34)	0.304
Control	324 / 83,532 (0.39)	538,177	6.02		1		1	
CCI scores = 1 (n = 18,815)								
Gout	30 / 4,169 (0.72)	28,353	10.60	2.43 (-1.47 to 6.29)	1.29 (0.85-1.96)	0.23	1.29 (0.90-1.85)	0.173
Control	83 / 14,646 (0.57)	101,583	8.17		1		1	
CCI scores ≥ 2 (n = 23,288)								
Gout	31 / 5,770 (0.54)	39,849	7.78	-3.02 (-6.63 to 0.56)	0.72 (0.49-1.07)	0.102	0.75 (0.54-1.04)	0.08
Control	128 / 17,518 (0.73)	118,360	10.80		1		1	

Abbreviation: IR, incidence rate; IRD, incidence rate difference; PY. person-year; OW, overlap weighting; IBD, Inflammatory bowel disease;
* Significance at P < 0.05
† Adjusted for age, sex, income, and region of residence, and CCI scores

Supplementary Table S5. Crude and adjusted hazard ratios (95% confidence interval) of gout for CD with subgroup analyses according to age, sex, income, region, and CCI scores

	N of event / N of total (%)	Follow-up duration (PY)	IR per 10000 (PY)	IRD (95% CI)	Hazard ratios for CD			
					Crude	P-value	Adjusted model with OW †	P-value
Total participants (n = 144,620)								
Gout	48 / 28,924 (0.17)	189,742	2.53	0.44 (-0.30 to 1.18)	1.21 (0.88-1.67)	0.249	1.18 (0.9-1.54)	0.224
Control	159 / 115,696 (0.14)	760,340	2.09		1		1	
Age < 45 years old (n = 85,645)								
Gout	27 / 17,129 (0.16)	119,006	2.27	0.65 (-0.19 to 1.49)	1.40 (0.91-2.18)	0.13	1.37 (0.95-1.98)	0.096
Control	77 / 68,516 (0.11)	476,190	1.62		1		1	
Age ≥ 45 years old (n = 58,975)								
Gout	21 / 11,795 (0.18)	70,736	2.97	0.08 (-1.32 to 1.49)	1.03 (0.64-1.66)	0.913	0.99 (0.68-1.46)	0.977
Control	82 / 47,180 (0.17)	284,150	2.89		1		1	
Male (n = 115,210)								
Gout	36 / 23,042 (0.16)	151,778	2.37	0.24 (-0.59 to 1.07)	1.11 (0.77-1.61)	0.567	1.09 (0.81-1.47)	0.58
Control	129 / 92,168 (0.14)	605,772	2.13		1		1	
Female (n = 29,410)								
Gout	12 / 5,882 (0.20)	37,964	3.16	1.22 (-0.44 to 2.88)	1.62 (0.83-3.17)	0.157	1.55 (0.87-2.75)	0.136
Control	30 / 23,528 (0.13)	154,568	1.94		1		1	
Low income group (n = 72,070)								
Gout	23 / 14,414 (0.16)	92,651	2.48	0.49 (-0.55 to 1.53)	1.25 (0.78-1.99)	0.355	1.20 (0.82-1.76)	0.354
Control	74 / 57,656 (0.13)	371,845	1.99		1		1	
High income group (n = 72,550)								
Gout	25 / 14,510 (0.17)	97,091	2.57	0.38 (-0.67 to 1.45)	1.18 (0.75-1.84)	0.477	1.16 (0.81-1.67)	0.425
Control	85 / 58,040 (0.15)	388,495	2.19		1		1	
Urban resident (n = 63,955)								
Gout	21 / 12,791 (0.16)	85,345	2.46	0.56 (-0.50 to 1.62)	1.30 (0.79-2.12)	0.303	1.31 (0.87-1.97)	0.201

Control	65 / 51,164 (0.13)	342,104	1.90		1		1	
Rural resident (n = 80,665)								
Gout	27 / 16,133 (0.17)	104,397	2.59	0.34 (-0.69 to 1.37)	1.15 (0.75-1.76)	0.524	1.10 (0.78-1.55)	0.601
Control	94 / 64,532 (0.15)	418,236	2.25		1		1	
CCI scores = 0 (n = 102,517)								
Gout	26 / 18,985 (0.14)	121,320	2.14	0.42 (-0.42 to 1.25)	1.24 (0.80-1.92)	0.331	1.25 (0.88-1.77)	0.218
Control	93 / 83,532 (0.11)	539,453	1.72		1		1	
CCI scores = 1 (n = 18,815)								
Gout	08 / 4,169 (0.19)	28,484	2.81	0.16 (-1.99 to 2.31)	1.06 (0.48-2.32)	0.894	1.07 (0.55-2.07)	0.852
Control	27 / 14,646 (0.18)	101,969	2.65		1		1	
CCI scores ≥ 2 (n = 23,288)								
Gout	14 / 5,770 (0.24)	39,938	3.51	0.23 (-1.84 to 2.30)	1.07 (0.58-1.98)	0.82	1.07 (0.63-1.83)	0.797
Control	39 / 17,518 (0.22)	118,918	3.28		1		1	

Abbreviation: IR, incidence rate; IRD, incidence rate difference; PY. person-year; OW, overlap weighting; CD, Crohn's disease;

* Significance at $P < 0.05$

† Adjusted for age, sex, income, and region of residence, and CCI scores

Supplementary Table S6. Crude and adjusted hazard ratios (95% confidence interval) of gout for UC with subgroup analyses according to age, sex, income, region, and CCI scores

	N of event / N of total (%)	Follow-up duration (PY)	IR per 10000 (PY)	IRD (95% CI)	Hazard ratios for UC			
					Crude	P-value	Adjusted model with OW †	P-value
Total participants (n = 144,620)								
Gout	94 / 28,924 (0.32)	189,460	4.96	0.01 (-1.11 to 1.13)	1.00 (0.80-1.25)	1	0.98 (0.82-1.18)	0.866
Control	376 / 115,696 (0.32)	758,986	4.95		1		1	
Age < 45 years old (n = 85,645)								
Gout	54 / 17,129 (0.32)	118,828	4.54	0.56 (-0.72 to 1.85)	1.14 (0.84-1.55)	0.387	1.14 (0.89-1.46)	0.294
Control	189 / 68,516 (0.28)	475,414	3.98		1		1	
Age ≥ 45 years old (n = 58,975)								
Gout	40 / 11,795 (0.34)	70,632	5.66	-0.93 (-3.02 to 1.16)	0.86 (0.61-1.21)	0.375	0.83 (0.64-1.09)	0.178
Control	187 / 47,180 (0.40)	283,572	6.59		1		1	
Male (n = 115,210)								
Gout	82 / 23,042 (0.36)	151,484	5.41	0.00 (-1.31 to 1.31)	1.00 (0.78-1.27)	1	0.99 (0.82-1.20)	0.913
Control	327 / 92,168 (0.35)	604,547	5.41		1		1	
Female (n = 29,410)								
Gout	12 / 5,882 (0.20)	37,976	3.16	-0.01 (-2.01 to 1.99)	1.00 (0.53-1.87)	0.988	0.94 (0.57-1.55)	0.809
Control	49 / 23,528 (0.21)	154,439	3.17		1		1	
Low income group (n = 72,070)								
Gout	54 / 14,414 (0.37)	92,469	5.84	1.10 (-0.51 to 2.70)	1.23 (0.91-1.67)	0.183	1.20 (0.93-1.54)	0.161
Control	176 / 57,656 (0.31)	371,248	4.74		1		1	
High income group (n = 72,550)								
Gout	40 / 14,510 (0.28)	96,991	4.12	-1.04 (-2.60 to 0.53)	0.80 (0.57-1.12)	0.196	0.79 (0.61-1.03)	0.086
Control	200 / 58,040 (0.34)	387,738	5.16		1		1	
Urban resident (n = 63,955)								
Gout	36 / 12,791 (0.28)	85,247	4.22	-1.32 (-3.04 to 0.41)	0.76 (0.53-1.09)	0.136	0.75 (0.57-0.99)	0.042*

Control	189 / 51,164 (0.37)	341,354	5.54		1		1	
Rural resident (n = 80,665)								
Gout	58 / 16,133 (0.36)	104,213	5.57	1.09 (-0.38 to 2.56)	1.24 (0.92-1.67)	0.15	1.22 (0.95-1.55)	0.114
Control	187 / 64,532 (0.29)	417,632	4.48		1		1	
CCI scores = 0 (n = 102,517)								
Gout	55 / 18,985 (0.29)	121,145	4.54	0.25 (-1.05 to 1.55)	1.06 (0.79-1.42)	0.711	1.05 (0.83-1.32)	0.674
Control	231 / 83,532 (0.28)	538,692	4.29		1		1	
CCI scores = 1 (n = 18,815)								
Gout	22 / 4,169 (0.53)	28,401	7.75	2.25 (-0.98 to 5.46)	1.40 (0.86-2.30)	0.177	1.39 (0.90-2.14)	0.139
Control	56 / 14,646 (0.38)	101,753	5.50		1		1	
CCI scores ≥ 2 (n = 23,288)								
Gout	17 / 5,770 (0.29)	39,914	4.26	-3.25 (-6.18 to -0.32)	0.57 (0.34-0.95)	0.032*	0.60 (0.39-0.91)	0.017*
Control	89 / 17,518 (0.51)	118,541	7.51		1		1	

Abbreviation: IR, incidence rate; IRD, incidence rate difference; PY. person-year; OW, overlap weighting; UC, Ulcerative colitis;
* Significance at P < 0.05
† Adjusted for age, sex, income, and region of residence, and CCI scores

Supplementary Figure S1. Kaplan–Meier analyses showed significantly higher cumulative incidence for IBD (log-rank p=0.0147) and CD (log-rank p=0.0277), but not for UC (log-rank p=0.1772).

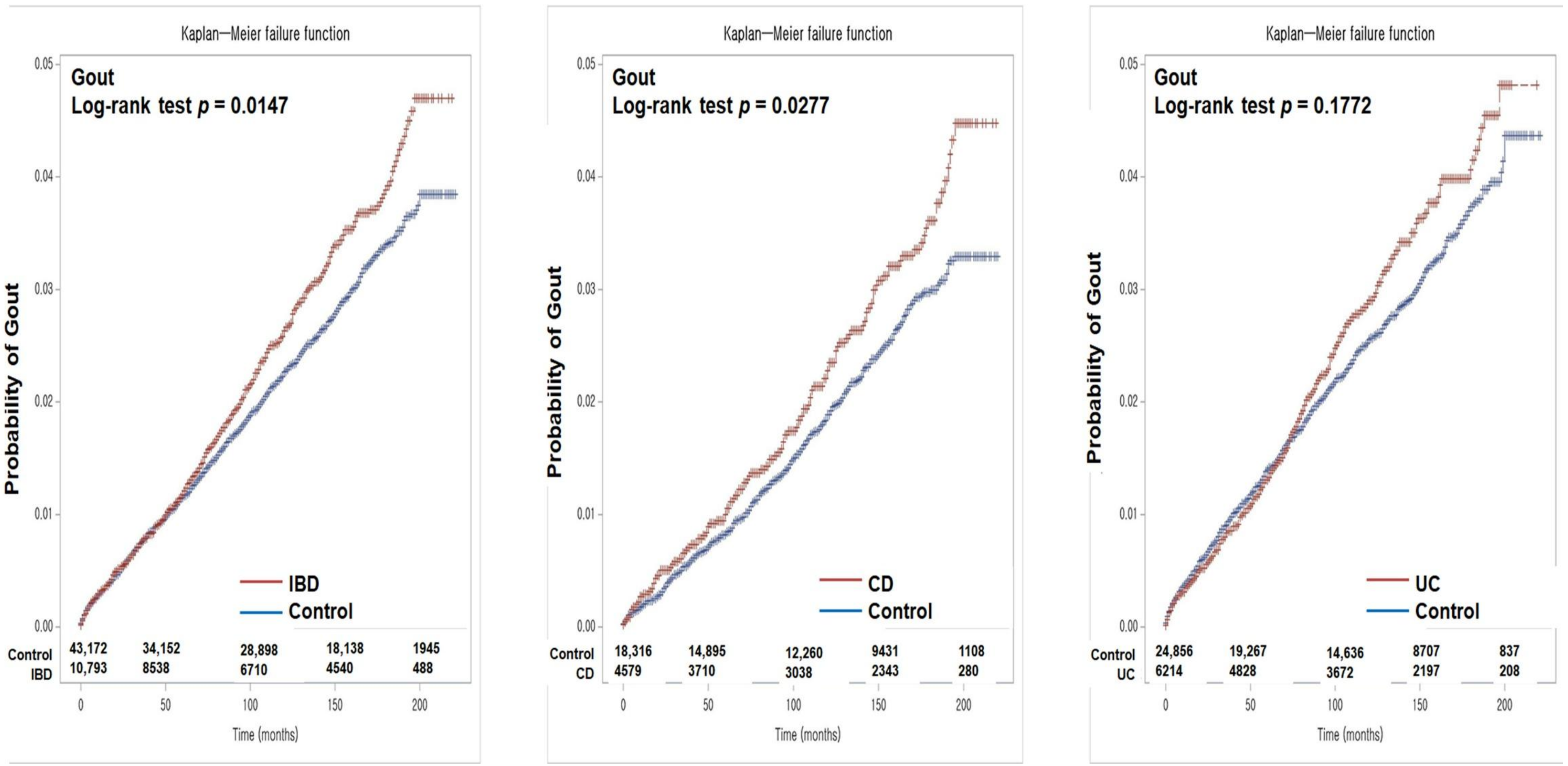

Supplementary Figure S2. Kaplan–Meier curves similarly showed no significant differences between groups [IBD (log-rank *p*=0.5190), CD (*p*=0.2487), or UC (*p*=0.9948)].

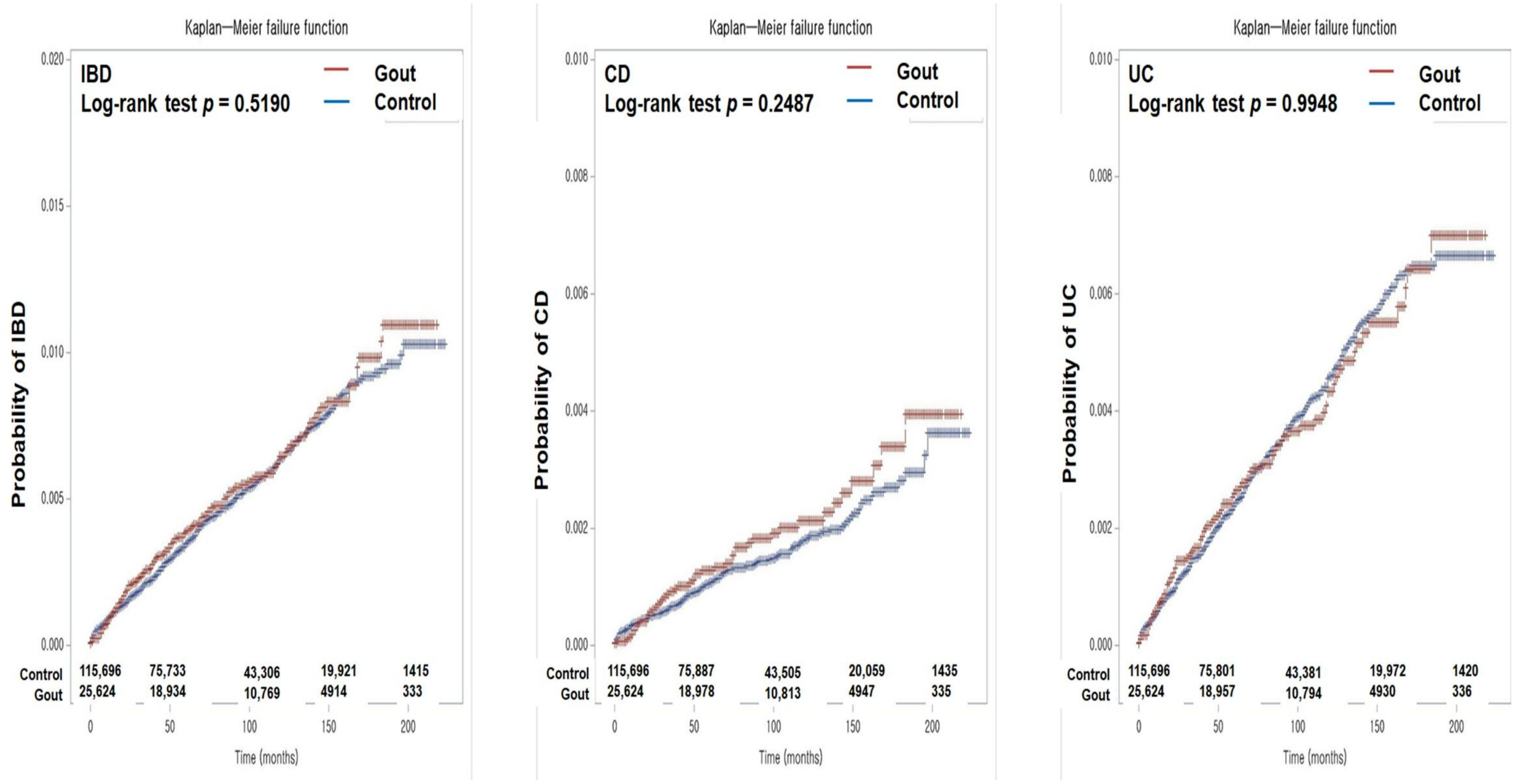

Supplement: Supplementary file 1 [file biomedicines-14-00613-s001.zip › biomedicines-4165845-supplementary.pdf]
